# Effects of γ-polyglutamic acid supplementation on alfalfa growth and rhizosphere soil microorganisms in sandy soil

**DOI:** 10.1038/s41598-024-57197-6

**Published:** 2024-03-18

**Authors:** Zhen Guo, Jian Wang, Tianqing Chen, Haiou Zhang, Xiandong Hou, Juan Li

**Affiliations:** 1 Institute of Land Engineering and Technology, Shaanxi Provincial Land Engineering Construction Group Co., Ltd., Xi’an, 710021 China; 2 Shaanxi Provincial Land Engineering Construction Group Co., Ltd., Xi’an, 710075 China; 3 Key Laboratory of Degraded and Unused Land Consolidation Engineering, Ministry of Natural Resources, Xi’an, 710021 China

**Keywords:** Soil nutrients, Plant growth, Bacterial community, γ-Polyglutamic acid, Mu Us Sandy land, Bacteria, Environmental microbiology, Microbiology, Ecology, Environmental sciences

## Abstract

This study aimed at exploring the effects of γ-polyglutamic acid on the growth of desert alfalfa and the soil microorganisms in the rhizosphere. The study examined the effects of varying concentrations of γ-polyglutamic acid (0%-CK, 2%-G1, 4%-G2, 6%-G3) on sandy soil, the research investigated its impact on the growth characteristics of alfalfa, nutrient content in the rhizosphere soil, and the composition of bacterial communities. The results indicated that there were no significant differences in soil organic matter, total nitrogen, total phosphorus, total potassium, and available phosphorus content among the G1, G2, and G3 treatments. Compared to CK, the soil nutrient content in the G2 treatment increased by 14.81–186.67%, showing the highest enhancement. In terms of alfalfa growth, the G2 treatment demonstrated the best performance, significantly increasing plant height, chlorophyll content, above-ground biomass, and underground biomass by 54.91–154.84%. Compared to the CK treatment, the number of OTUs (operational taxonomic units) in the G1, G2, and G3 treatments increased by 14.54%, 8.27%, and 6.84%, respectively. The application of γ-polyglutamic acid altered the composition and structure of the bacterial community, with *Actinobacteriota*, *Proteobacteria*, *Chloroflexi*, *Acidobacteriota*, and *Gemmatimonadota* accounting for 84.14–87.89% of the total bacterial community. The G2 treatment significantly enhanced the diversity and evenness of soil bacteria in the rhizosphere. Redundancy analysis revealed that organic matter, total nitrogen, total potassium, moisture content, and pH were the primary factors influencing the structure of bacterial phyla. At the genus level, moisture content emerged as the most influential factor on the bacterial community. Notably, moisture content exhibited a strong positive correlation with Acidobacteriota, which in turn was positively associated with indicators of alfalfa growth. In summary, the application of γ-polyglutamic acid at a 4% ratio has the potential for improving sandy soil quality, promoting plant growth, and regulating the rhizosphere microbial community.

## Introduction

γ-Polyglutamic acid, a polymer consisting of the amino acid glutamic acid, has attracted considerable attention from researchers due to its exceptiona water and moisture retention properties, along with its capacity to enhance organic matter^1^. Incorporating γ-polyglutamic acid into soil has been demonstrated as an effective approach to improve soil texture and water retention ability^2^. This application results in increased soil organic matter content, enhanced soil structure, and promotion of microbial activity in the soil^3^. The utilization of γ-polyglutamic acid exhibits promising potential in the restoration of sandy soil ecosystems and stimulation of plant growth, thereby playing a critical role in enhancing the overall functionality and productivity of sandy lands.

Alfalfa (*Medicago sativa* L.) is a significant leguminous forage crop that plays a crucial role in breeding and soil conservation. It possesses the capability to fix nitrogen gas in the soil, enhance soil texture and fertility, and have a beneficial influence on the soil microbial community^3^. However, alfalfa growth is limited to sandy soils, which typically have low water retention capacity and organic matter content^4^. These factors pose challenges for the normal growth and development of alfalfa^5^. However, research has shown that the use of γ-polyglutamic acid can notably improve the growth of alfalfa in sandy environments^6^. Compared to the control group, alfalfa plants supplemented with γ-polyglutamic acid showed significant enhancements in biomass, root structure, and chlorophyll content^6^. These findings indicate the promising potential of γ-polyglutamic acid for enhancing sandy soil quality^4–6^. Compared to conventional fertilizers, the utilization rate of nitrogen, phosphorus, and potassium fertilizer in eggplant increased by 18%, 16%, and 7% when using γ-polyglutamic acid as a fertilizer^7^. The application of γ-polyglutamic acid to crops such as rape, alfalfa, cabbage, tomato, corn, and wheat has demonstrated increased production and reduced fertilizer usage^8^. Additionally, research has shown that γ-polyglutamic acid can enhance soil water retention capacity, particularly benefiting the growth of plants like alfalfa in sandy soil^9^. This study contributes valuable insights into the positive effects of γ-polyglutamic acid on soil structure and water distribution^7–9^. Multiple research studies have explored the impact of γ-polyglutamate on soil microbial communities^10,11^. These studies indicate that the application of γ-polyglutamate can enhance the diversity and activity of soil microorganisms, potentially attributed to increase soil organic matter and improve microbial habitats^10^. Research has indicated that the utilization of γ-polyglutamic acid fermentation broth and concentrated γ-polyglutamic acid granule fertilizer products can enhance the richness, diversity, and evenness of soil microbial communities^11^. These results underscore the multiple advantages of γ-polyglutamic acid as a soil supplement. In addition to enhancing soil physical characteristics, it also plays a role in the rehabilitation and resilience of soil ecosystems^12,13^.

Previous research has shown the beneficial effects of γ-polyglutamic acid on crop growth conditions, water and fertilizer utilization^14,15^. However, additional assessment is needed to understand its potential application in sandy land. This study aims to examine the impact of γ-polyglutamic acid supplementation on alfalfa growth and the function of rhizosphere soil microbes.The study aims to explore the potential of γ-polyglutamic acid in remediating sandy soil and enhancing agricultural production. It seeks to provide scientific evidence and practical recommendations for addressing the issue of sandy soil. The research on γ-polyglutamic acid is expected to make a significant contribution to improving soil quality, promoting plant growth, protecting the ecological environment, and enhancing agricultural sustainability.

## Materials and methods

### Test materials

The soil sample utilized in this study was aeolian sandy soil obtained from Xiaojihan Village, located in Yulin City, Shaanxi Province, in northwest China. The sample was collected at a depth of 0–30 cm. The sampling site is subject to a temperate continental monsoon climate, characterized by distinct four seasons, significant daily temperature fluctuations, a short frost-free period, an average annual temperature of 8 ℃, an average annual precipitation of approximately 400 mm, and an average annual sunshine duration ranging from 2593.5 to 2914.4 h. The collected aeolian sandy soil was separated from debris and residual root systems. It was then air dried and screened using a 1 mm sieve. The basic physical and chemical properties of the aeolian sandy soil are as follows: organic matter content of 2.22 g kg^−1^, total nitrogen content of 0.12 g kg^−1^, bulk density of 1.60 g cm^−3^, total phosphorus content of 0.09%, total potassium content of 1.56%, pH of 8.8, SiO_2_ content of 77.12%, FeO content of 2.06%, CaO content of 2.18%, and K_2_O content of 2.44%. The agricultural γ-polyglutamic acid utilized in the experiment was a white powder sourced from Shandong Freida Biotechnology Co., Ltd. This particular γ-polyglutamic acid had a particle size of 0.149 mm and a molecular weight of 700 kD. With an effective content of 30%, it serves as an environmentally friendly degradable polymer material.

### Experimental design

A pot experiment was conducted in this study to investigate the effects of γ-polyglutamic acid on plant growth. A large pot container with a diameter of 20 cm and a height of 30 cm was selected. Prior to filling the pot with soil, γ-polyglutamic acid was mixed into the aeolian sandy soil at mass ratios of 0% (CK), 2% (G1), 4% (G2), and 6% (G3). The γ-polyglutamic acid was thoroughly mixed with the soil to match the bulk density of the field soil. Subsequently, the soil was packed into the pot container to the same bulk density, and the pot tray was filled with water to raise the water level through suction, ensuring uniform soaking of the topsoil in the pot. Subsequently, ten germinated alfalfa seeds were carefully placed in the topsoil layer of each pot and covered with a 5 mm layer of soil to ensure adequate moisture retention. The experiment was carried out with three replicates for each treatment, resulting in a total of twelve plants. During the growth phase of alfalfa, a ternary compound fertilizer with a composition of N-P-K = 15-15-15 was consistently applied. Watering was scheduled every 10 days, with 1 g of fertilizer dissolved in 200 mL of water for each pot irrigation. This fertilization and irrigation process was repeated five times.

### Determination of agronomic traits

The growth period of alfalfa usually spans approximately 50 days. Upon reaching the harvest period, the plants were removed from the ground and their height was assessed with a tape measure. Chlorophyll levels in the plants were determined with a chlorophyll meter (SPAD-502Plus, Qingdao, CHN), while the weight of both above-ground and underground dry matter was measured using an electronic balance.

### Soil sample collection and determination

On May 30, 2022, three plants were randomly selected in each treatment, with the main root of alfalfa as the center. The sampling area had a radius of 5 cm and a depth of 10 cm. Soil samples were excavated, mixed, cleaned of debris, and spread into squares. The soil was divided into four parts by diagonals, with two diagonal parts collected as one soil sample. The remaining part was backfilled. A portion of the collected soil was air-dried for physical and chemical property analysis, while another part was stored at – 80 ℃ for rhizosphere microorganism analysis.

Soil pH was measured using a pH meter, while soil organic matter (SOM) was determined using the potassium dichromate and external heating method. The Kjeldahl method was employed to analyze soil total nitrogen (TN), the molybdenum-antimony resistance colorimetric method was utilized for soil total phosphorus (TP), and the flame photometer was used for soil total potassium (TK). Soil available phosphorus (AP) was determined through NaHCO_3_ extraction using the molybdenum-antimony resistance colorimetric method, soil available potassium (AK) was determined through CH_3_COONH_4_ extraction using flame atomic photometry, and soil moisture content (MC) was measured by drying method^16–18^. The height of alfalfa was measured using a vernier caliper (Mitutoyo 514-106, JPN), chlorophyll was measured using a chlorophyll analyzer (SPAD-502Plus, CH), and biomass was measured using a one percent electronic balance (YP-2, CH).

Bacterial DNA samples were extracted using the OMEGA Soil DNA Kit (M5635-02) (Omega Bio-Tek, Norcross, GA, USA) and stored at – 20 ℃ before further analysis. The total DNA concentration and mass extracted were determined by NanoDrop NC2000 spectrophotometer (Thermo Fisher Scientific, Waltham, MA, USA) and agar-gel electrophoresis, respectively^19^. The bacterial 16S rRNA (V3 + V4) region primers: forward primer 5′-ACTCCTACGGGAGGCAGCA-3′, reverse primer 5′-GGACTACHVGGGTWTCTAAT-3′^20^. PCR products were quantified on a Microplate reader (BioTek, FLx800) using the Quant-iT PicoGreen dsDNA Assay Kit. Samples were mixed according to the required data amount for each sample. The library was constructed using Illumina's TruSeq Nano DNA LT Library Prep Kit, followed by inspection and quantification. A 1 μL library was taken for quality inspection using the Agilent High Sensitivity DNA Kit on the Agilent Bioanalyzer machine. A qualified library should exhibit a single peak without any contamination^21^. Quant-iT PicoGreen dsDNA Assay Kit was used to quantify the library on Promega QuantiFluor, and qualified libraries should have a calculated concentration of more than 2 nmol L^−1^. Qualified libraries were sequenced 2 × 250 bp double-ended using the NovaSeq 6000 SP Reagent Kit (500 cycles) on the Illumina NovaSeq machine^22^.

### Data analysis

The data was compiled using Microsoft Excel 2016 software to analyze the notable variations in alfalfa plant height, chlorophyll levels, and above-ground and subsurface dry matter mass. This analysis was conducted utilizing the minimum significant difference (LSD) method in SPSS 20.0 software with One-way ANOVA. The dilution curve was selected at the OTU (operational taxonomic units) classification level with 97% similarity, the coverage index under different random samples was calculated with mothur, and the graph was made with R language tool. Venn diagrams are a valuable tool for quantifying the overlap and unique features of operational taxonomic units (OTUs) across multiple sample sets in R language (version 3.3.1). The bacterial community composition at the phylum level was analyzed using R tools. A circular diagram representing the relationship between samples and species at the genus level was generated using Circos-0.67-7. Beta diversity distance matrices were computed using Qiime, while non-metric multidimensional scaling analysis (NMDS) analysis was conducted in R language (version 2.4.3). One-way ANOVA was utilized to assess differences in diversity indices and species composition. The vegan package in R language (version 2.4.3) facilitated redundancy analysis (RDA) and visualization. Correlation analysis for heatmap generation was performed using R language (version 3.3.1).

### Ethical approval

All procedures with plants were conducted in accordance with the guidelines and regulations.

## Results

### Nutrient content of alfalfa rhizosphere soil

The γ-polyglutamic acid has a significant impact on the physical and chemical properties of sandy soil, as shown in Table 1. Initially, there were no significant differences in the content of SOM, TN, TP, TK, and AP among the G1, G2, and G3 treatments, with no significant variance between G1 and the CK treatment. However, compared to the CK treatment, the SOM content increased significantly by 86.42% and 62.91% (P < 0.05) under the G2 and G3 treatments, respectively. Additionally, the TP content showed a significant increase of 40.59% and 28.71%, while the TK content increased significantly by 14.81% and 13.39% in the G2 and G3 treatments. The content of TN and AP significantly increased compared to CK treatment only under G2 treatment, with an increase of 186.67% and 35.38%. AK content showed a significant gradient difference across treatments, with G2 > G3 > G1 > CK, and G2 treatment exhibited the highest increase. pH levels remained similar between CK, G1, and G3 treatments, but there was a significant 9.71% decrease observed in G2 treatment. Changes in MC content were also significant, with G1, G2, and G3 treatments showing increases of 41.93%, 77.02%, and 54.66% compared to CK treatment.

**Table 1:** Effects of addition of γ-polyglutamic acid on nutrient characteristics of sandy soil.

Treatments	SOM (g kg^−1^)	TN (g kg^−1^)	TP (g kg^−1^)	TK (g kg^−1^)	AP (mg kg^−1^)	AK (mg kg^-−1^)	pH	MC (%)
CK	3.02 ± 0.11 b	0.15 ± 0.03 b	1.01 ± 0.04 b	17.55 ± 1.32 b	3.90 ± 0.57 b	45.55 ± 3.20 d	8.75 ± 1.16 a	3.22 ± 0.11 c
G1	4.15 ± 0.50 ab	0.30 ± 0.05 ab	1.26 ± 0.15 ab	18.41 ± 2.02 ab	4.36 ± 1.31 ab	58.26 ± 1.57 c	8.49 ± 0.98 a	4.57 ± 0.14 b
G2	5.63 ± 1.14 a	0.43 ± 0.04 a	1.42 ± 0.03 a	20.15 ± 2.11 a	5.28 ± 1.05 a	97.45 ± 3.56 a	7.90 ± 1.24 b	5.70 ± 0.56 a
G3	4.92 ± 0.23 a	0.36 ± 0.10 ab	1.30 ± 0.31 a	19.90 ± 1.95 a	4.56 ± 0.04 ab	72.13 ± 2.90 b	7.96 ± 1.22 ab	4.98 ± 0.32 ab

CK, G1, G2 and G3 represent 0, 2%, 4% and 6% γ-polyglutamic acid additions in aeolian sandy soil, respectively. SOM represents organic matter content, TN represents total nitrogen content, TP represents total phosphorus content, TK represents total potassium content, AP represents available phosphorus content, AK represents available potassium content, and MC represents soil moisture content. Mean ± standard deviation, lowercase letters indicate significant differences between different treatments (P < 0.05).

### Growth characteristics of alfalfa

The growth and development parameters of alfalfa, including plant height (AH), above ground biomass (AB), underground biomass (UB), and leaf chlorophyll content, were significantly influenced by supplementation with γ-glutamic acid. The variations in these parameters were in line with the rise in γ-glutamic acid concentration (Table 2). With the increment of γ-glutamic acid concentration, the AH seedling increased first and then decreased. G1 and G2 treatments were found to enhance the growth of alfalfa, whereas G3 treatments were observed to hinder its growth. The levels of AB, UB, and chlorophyll content exhibited a consistent pattern with the application of γ-glutamic acid. In comparison to CK treatment, the AH, chlorophyll, AB, and UB levels significantly rose by 154.08%, 54.91%, 141.84%, and 154.84%, respectively, under the G2 treatment.

**Table 2:** Effects of γ-polyglutamic acid supplementation on growth of alfalfa.

Treatments	AH (cm)	Chlorophyll (SPAD)	AB (g)	UB (g)
CK	6.12 ± 1.05 b	28.54 ± 4.18 b	0.98 ± 0.02 b	0.31 ± 0.04 b
G1	13.56 ± 3.02 a	37.63 ± 5.16 ab	1.67 ± 0.23 ab	0.45 ± 0.03 b
G2	15.55 ± 2.55 a	44.21 ± 5.01 a	2.37 ± 0.18 a	0.79 ± 0.12 a
G3	4.25 ± 0.32 b	25.54 ± 5.74 b	0.48 ± 0.03 b	0.20 ± 0.05 c

CK, G1, G2 and G3 represent 0, 2%, 4% and 6% γ-polyglutamic acid additions in aeolian sandy soil, respectively. AH stands for alfalfa plant height, AB stands for aboveground biomass, UB stands for underground biomass. Mean ± standard deviation, lowercase letters indicate significant differences between different treatments (P < 0.05).

### OTUs quantity and coverage of soil bacteria

**Figure 1:**
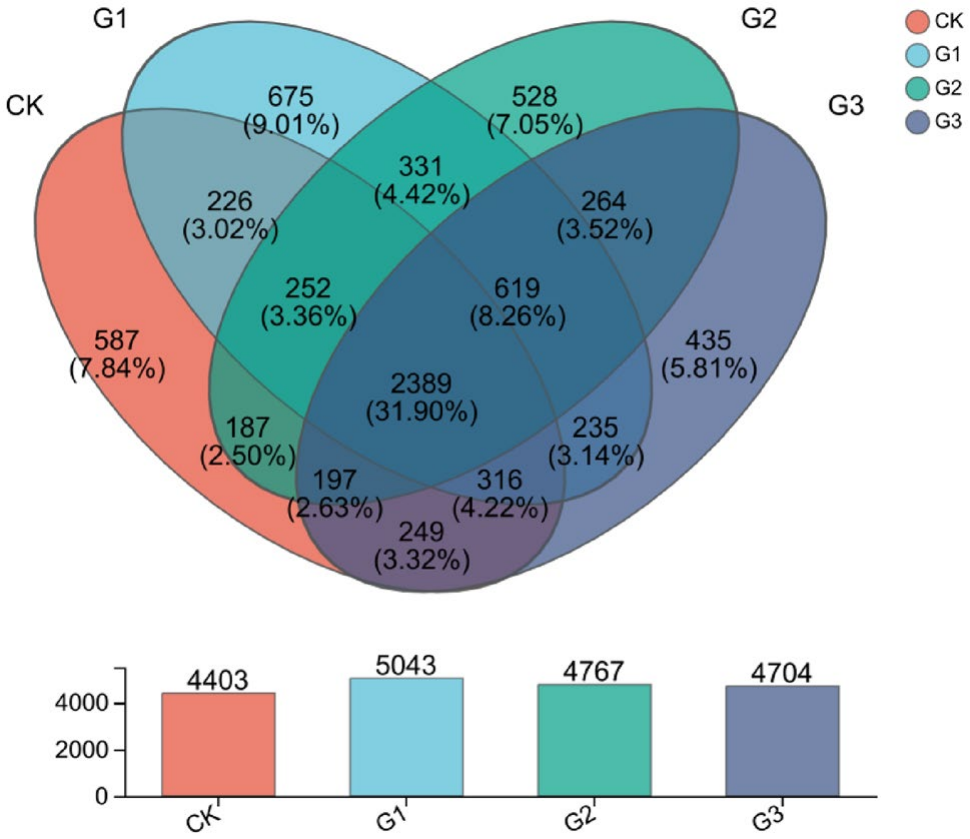
Number of bacterial OTUs species under different treatments. Different colors represent different groups, and the bar chart shows the total number of species.

**Figure 2:**
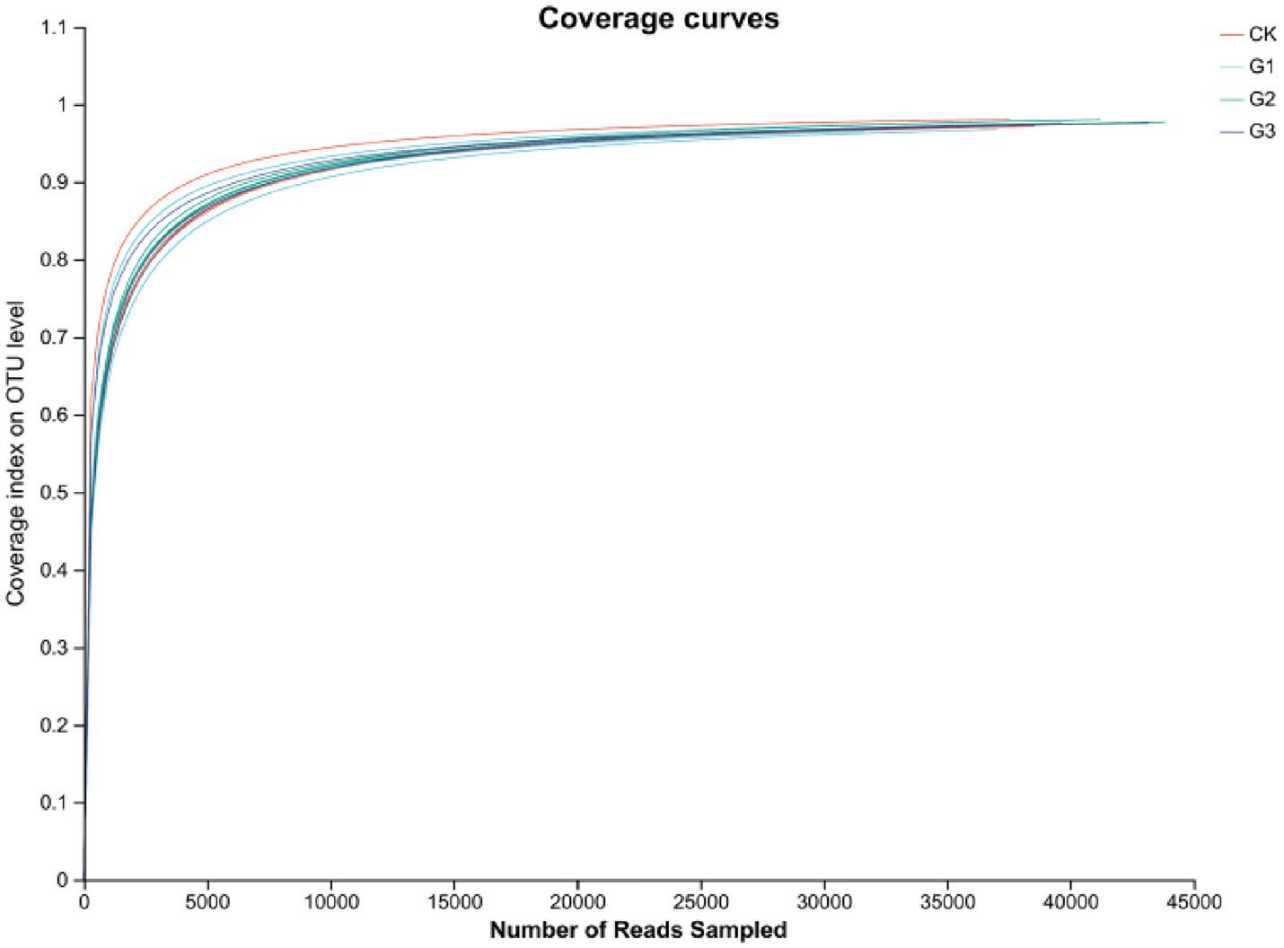
Species dilution curves under different treatments. The horizontal axis represents the amount of randomly selected sequencing data, while the vertical axis represents the coverage index.

The addition of γ-polyglutamic acid can increase the number of bacterial species in sandy soil. Compared to CK, the number of OTUs processed by G1, G2 and G3 increased by 14.54%, 8.27% and 6.84%, respectively (Fig. 1). There was some species similarity between each treatment, and 2389 species were common for all the four treatments. There were a total of 3183, 3025, and 3151 species between CK and G1, G2, G3, respectively. There were 3591 species shared between G1 and G2, and 3559 species shared between G1 and G3. There were 3469 species shared between G2 and G3. The species dilution curve tends to plateau as the sequencing data approaches saturation, effectively capturing a high proportion of species in the rhizosphere soil microbial community of alfalfa, achieving a coverage rate of 97.72%. The data was deemed relatively reliable (Fig. 2).

### Bacterial community composition of alfalfa rhizosphere soil

**Figure 3:**
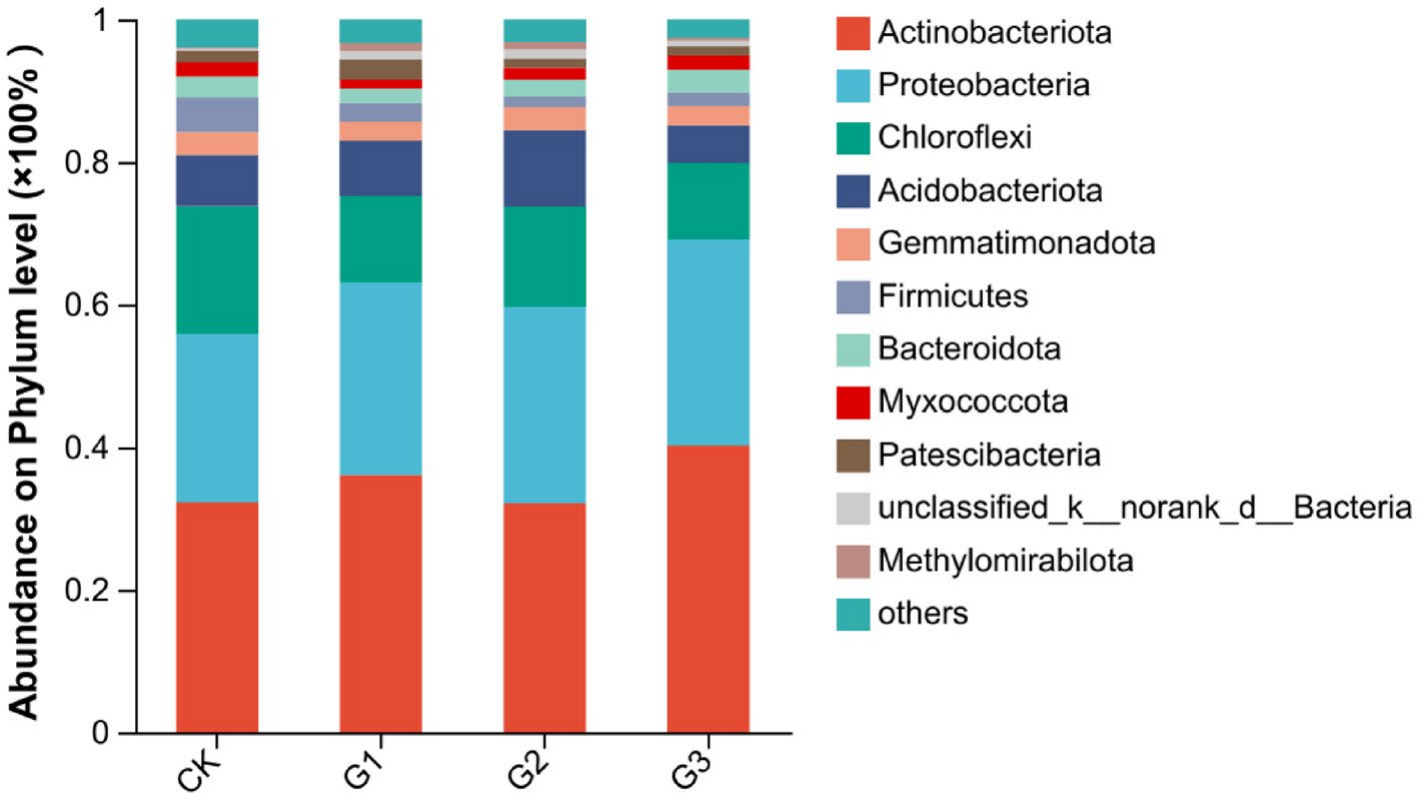
Composition of soil bacterial community at Phylum level under γ-polyglutamic acid supplementation. CK, G1, G2 and G3 represent 0, 2%, 4% and 6% γ-polyglutamic acid additions in aeolian sandy soil, respectively.

The addition of γ-polyglutamic acid had a significant impact on the community composition at the bacterial Phylum level. At the Phylum level, the top five bacteria showing higher abundance were *Actinobacteriota*, *Proteobacteria*, *Chloroflexi*, *Acidobacterota*, and *Gemmatimonadota*, accounting for 84.14% to 87.89% of the total bacterial community (Fig. 3). Compared to CK treatment, the abundance of *Actinobacteriota* increased by 12.07% and 24.74% under G1 and G3 treatments, respectively, while G2 treatment decreased by 0.16%, showing no difference from CK treatment. The abundance of *Proteobacteria* increased with the increase of γ-polyglutamic acid addition, with an increase between 14.35 and 22.75% compared to CK, with the highest increment observed in G3 treatment. The abundance of *Chloroflexi* decreased due to the addition of γ-polyglutamic acid, with a decrement of 32.28%, 21.61%, and 40.17% for G1, G2, and G3 treatments, respectively. There was no significant difference in the abundance of *Acidobacterota* between G1 and CK. The abundance of G2 treatment increased by 49.16% compared to CK, while the abundance of G3 treatment decreased by 27.02% compared to CK. Additionally, the abundance of *Gemmatimonadota* slightly decreased under G1 and G3 treatments, although this difference was not statistically significant. Compared to CK, the abundance of non-dominant bacteria *Firmicutes* decreased by 46.06%, 70.30%, and 61.41% under G1, G2, and G3 treatments, with the G2 treatment showing the greatest decrease.

**Figure 4:**
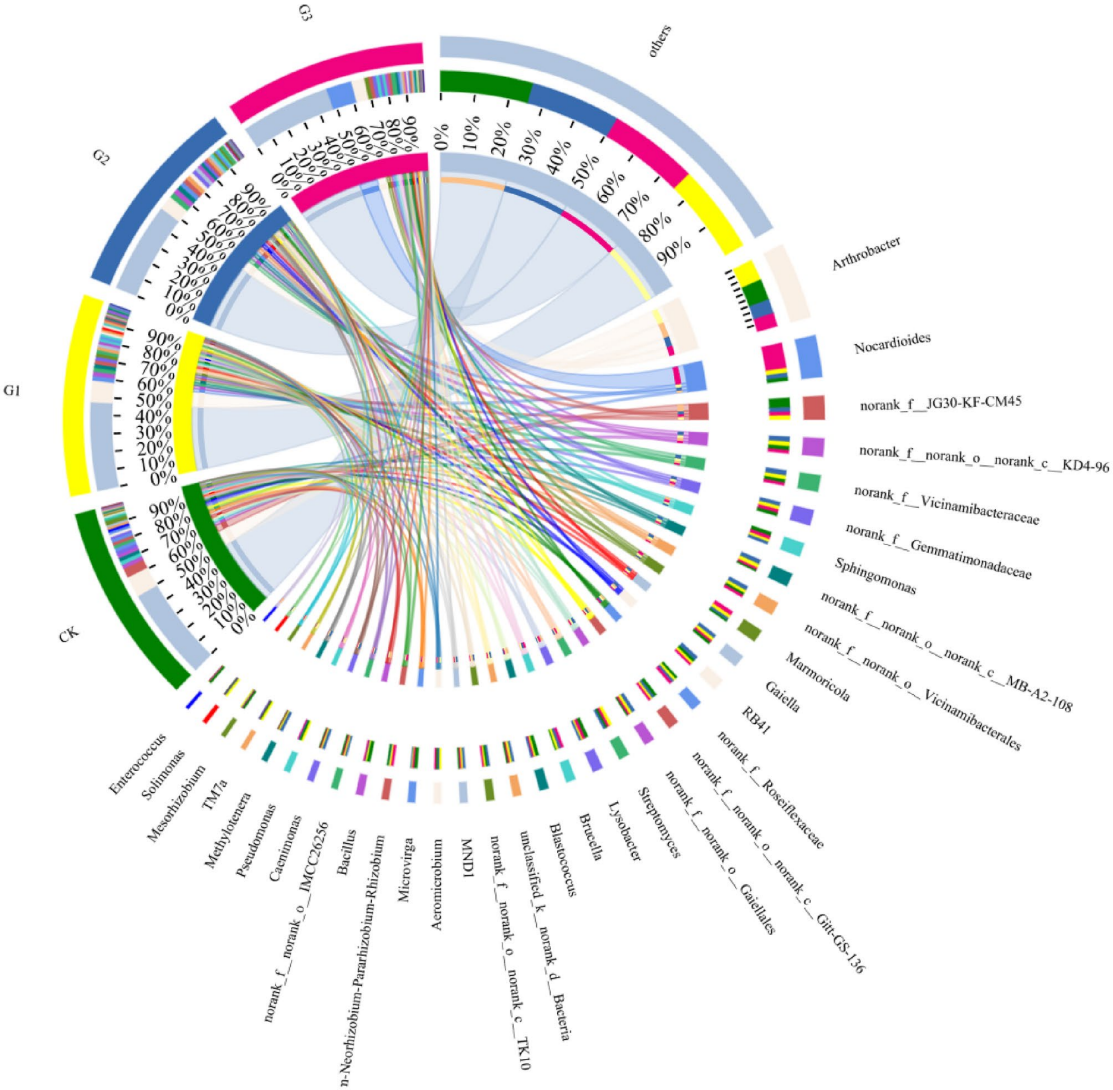
Effect of γ-polyglutamic acid on soil bacterial community composition at Genus level. CK, G1, G2 and G3 represent 0, 2%, 4% and 6% γ-polyglutamic acid additions in aeolian sandy soil, respectively.

**Figure 5:**
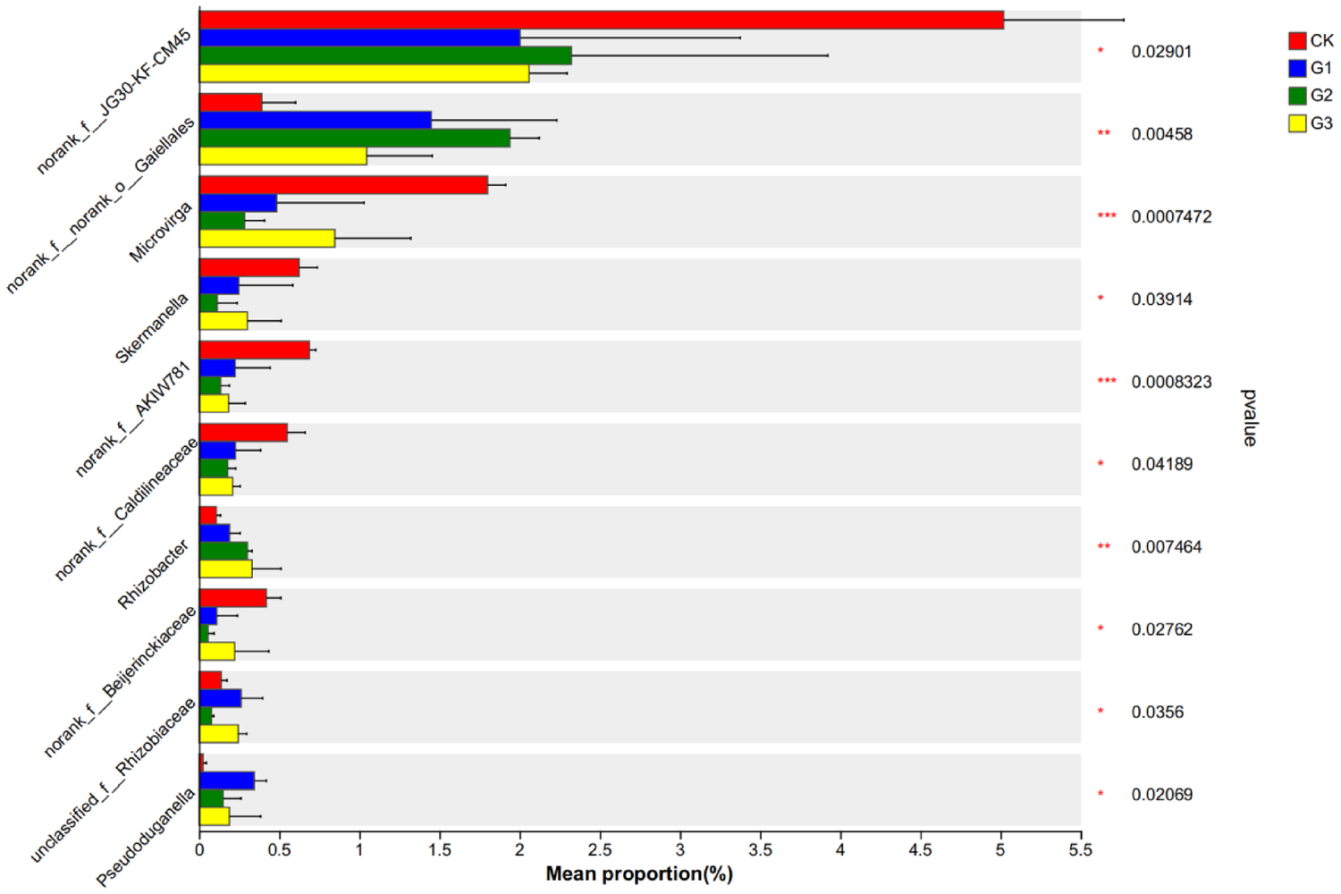
Intergroup differences in bacterial communities at the genus level. The Y-axis represents species names at a taxonomic level, the X-axis represents the average relative abundance of species in different groups, and the columns of different colors represent different groups. The far right is the P value, * means P < 0.05, ** means P < 0.01, *** means P < 0.001.

Under the Genus level classification mode, the soil bacterial community changed significantly, and the abundance of unknown bacterial genera is relatively high. *Arthroactor*, *Nocardioides*, *norank_ F__ JG30-KF-CM45*, *norank_ F__ Norank_ O__ Norank_ C__ KD4-96* and *norank_f__Vicinamibacteraceae* were the most abundant species five species, accounting for 21.70%-25.27% of the total bacterial community. Compared to CK treatment, the abundance of *Arthrobacter* decreased by 32.71% and 37.84% under G2 and G3 treatments, respectively (Fig. 4). Compared to CK treatment, the abundance of *Nocardioides* in G1, G2, and G3 treatments increased by 53.04%, 46.34%, and 729.88%, respectively. Various bacterial communities display a certain level of diversity at the Genus level. Notably, *norank_f__JG30-KF-CM45*, *Skermanella*, *norank_f__Caldilineaceae*, *norank_f__Beijerinckiaceae*, *unclassified_f__Rhizobiaceae*, and *Pseudoduganella* showed significantly different abundance levels at a 5% significance level. (Fig. 5).

### Soil bacterial diversity index

**Figure 6:**
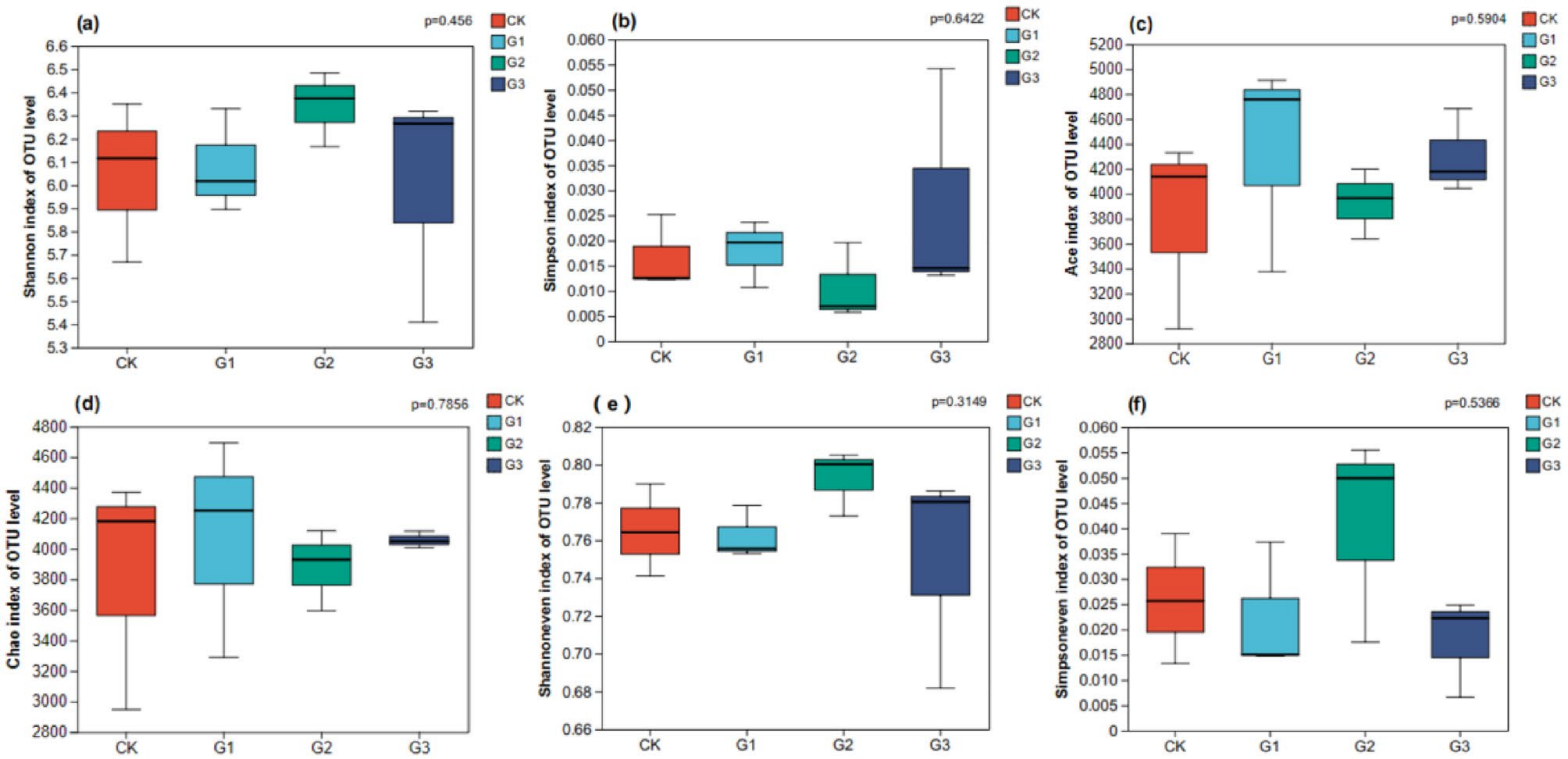
Bacterial diversity index of alfalfa rhizosphere soil under different treatments. (**a**) Is the Shannon index, (**b**) is the Simpson index, (**c**) is the Ace index, (**d**) is the Chao index, (**e**) is the Shannoneven index, (**f**) is the Simpsoneven index. CK, G1, G2 and G3 represent 0, 2%, 4% and 6% γ-polyglutamic acid additions in aeolian sandy soil, respectively.

The addition of γ-polyglutamic acid did not have a significant impact on soil bacterial diversity index, richness index, and evenness index. However, there were notable changes in values as shown in Fig. 6. The Shannon and Simpson indices are indicators of bacterial diversity. Compared to CK, the Shannon indices of treatments G2 and G3 increased by 6.45% and 6.28% respectively (Fig. 6a), while the Simpson indices of treatments G1 and G3 increased by 25.00% and 16.67% respectively (Fig. 6b). Ace and Chao index represent bacterial richness. Compared to CK, Ace and Chao index of G1 treatment showed an increasing trend, increasing by 14.96% and 1.67% respectively (Fig. 6c and Fig. 6d). Shannoneven and Simpsoneven indices represent bacterial evenness. Compared to CK, the Shannoneven indices of G2 and G3 treatments increase by 5.26% and 2.63%, respectively (Fig. 6e), and the Simpsoneven indices of G2 treatment increase by 92.30% (Fig. 6f). G2 treatment promoted the increase of bacterial diversity and uniformity, while G1 treatment promoted the increase of bacterial abundance.

### Similarity of soil bacterial communities

**Figure 7:**
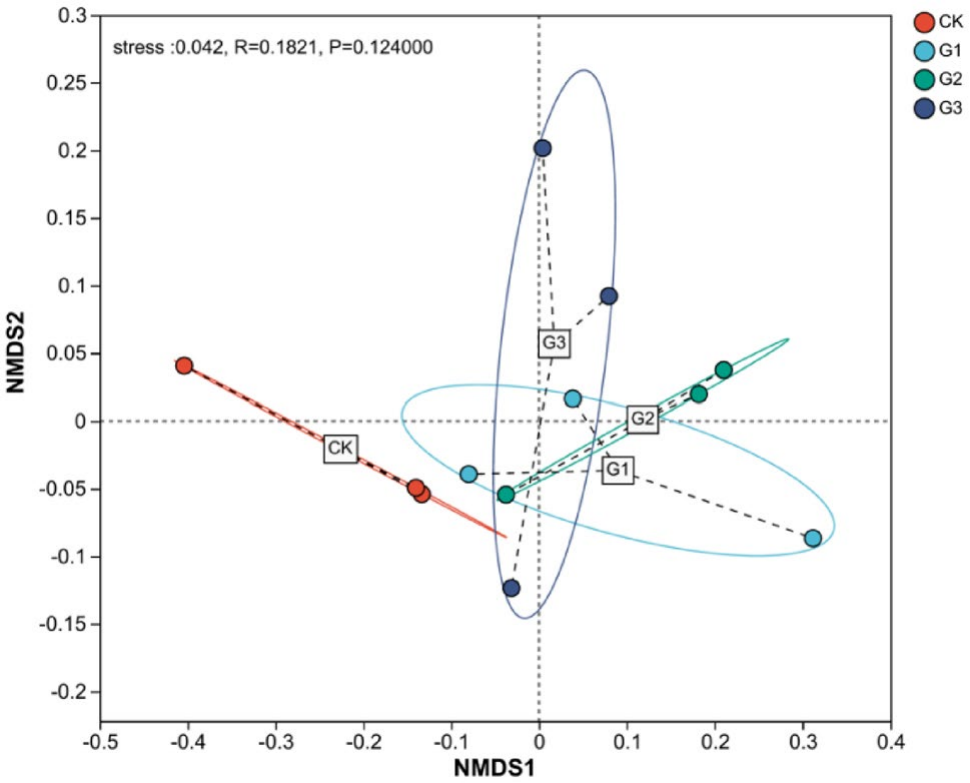
Non-metric multidimensional scaling analysis (NMDS) of soil bacterial communities. Stress indicates whether the analysis result of NMDS is good or bad. When stress < 0.05, it is representative. CK, G1, G2 and G3 represent 0, 2%, 4% and 6% γ-polyglutamic acid additions in aeolian sandy soil, respectively.

The addition of γ-polyglutamic acid to sandy soil significantly influenced the bacterial community structure in the rhizosphere soil of alfalfa. The stress value obtained in this study was 0.042, indicating high representativeness of the NMDS analysis results. NMDS analysis revealed that treatments G1, G2, and G3 exhibited high similarity in the composition of soil bacterial communities (Fig. 7). Among the treatments, G1 and G2 exhibited closer proximity in distance and higher similarity. The CK treatment was positioned to the left of the central axis of NMDS1, whereas the G1, G2, and G3 treatments were situated to the right, suggesting notable disparities in community structure between the CK treatment and the others.

### Redundancy analysis (RDA) of soil properties and bacterial communities

**Figure 8:**
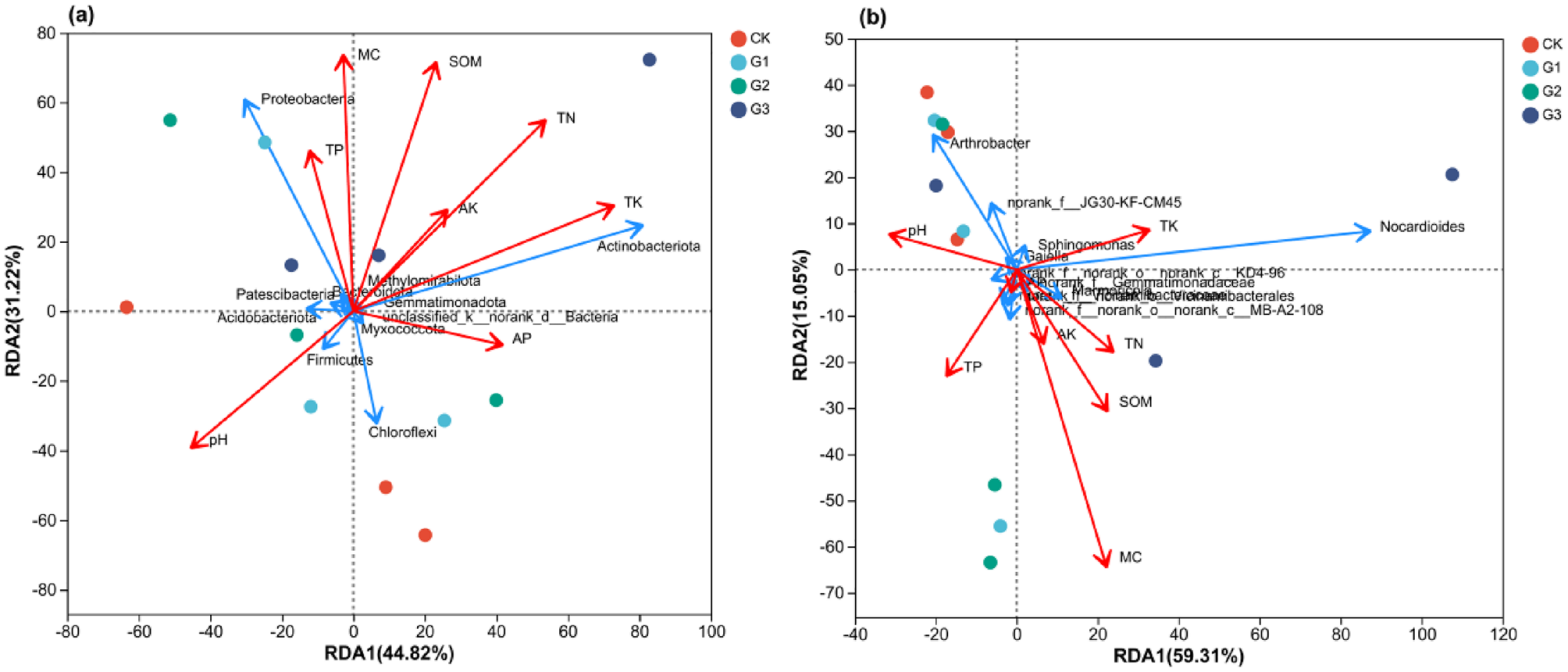
Redundancy analysis (RDA) of soil properties and bacterial communities based on phylum and genus levels. CK, G1, G2 and G3 represent 0, 2%, 4% and 6% γ-polyglutamic acid additions in aeolian sandy soil, respectively. SOM represents organic matter content, TN represents total nitrogen content, TP represents total phosphorus content, TK represents total potassium content, AP represents available phosphorus content, AK represents available potassium content, and MC represents soil moisture content. Environmental factors are indicated by red arrows, and bacterial species are indicated by blue arrows.

The redundancy analysis (RDA) conducted on the top 11 species and soil properties at the phylum and genus levels provides insights into the influence of environmental factors on microbial communities. It helps in understanding the relationship between environmental factors, samples, and microbial communities. The results show that the RDA1 and RDA2 axes collectively explain 76.04% and 74.36% of the variations in the taxonomic structure of bacterial phyla and genera, respectively. At the level of bacterial phylum classification, SOM, TN, TK, MC, and pH had the greatest impact on bacterial communities, followed by TP, AP, and AK. *Proteobacteria* showed a positive correlation with TP, MC, SOM, TN, and AK. *Actinobacteriota* exhibited a positive correlation with TK, AP, AK, TN, SOM, MC, and TP, while *Chloroflexi* was only positively correlated with pH and AP (Fig. 8a). Moving to the classification level of bacterial genus, MC had the most significant effect on the bacterial community, followed by SOM, pH, TK, and TP, while AP and AK had the least impact. *Arthrobacter* was positively associated with pH, and *Nocardioides* showed positive correlations with TK, TN, SOM, AK, and MC (Fig. 8b).

### Relationship between bacterial communities and soil indexes and alfalfa growth

**Figure 9:**
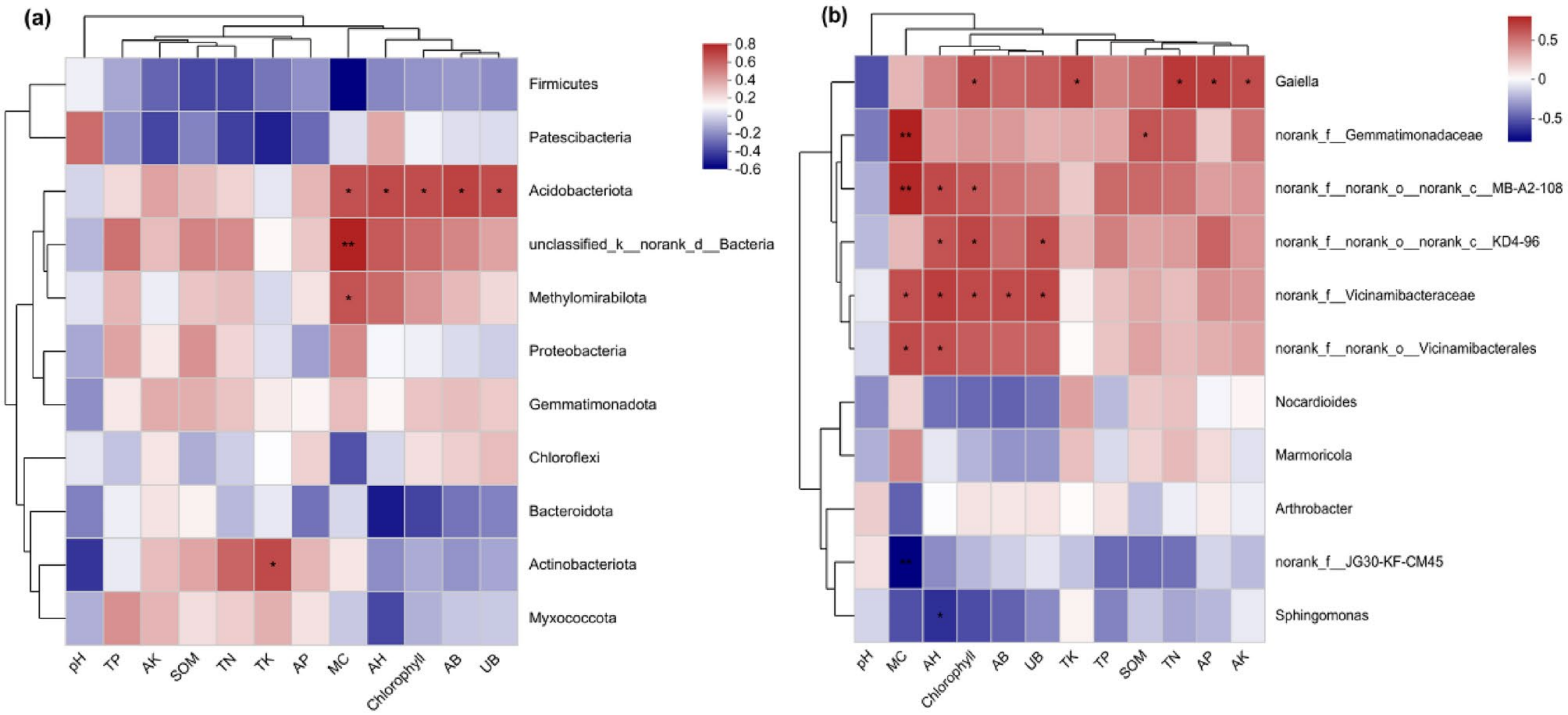
Pearson correlation between phylum (**a**) and genus (**b**) level bacterial communities and soil properties and alfalfa growth indices. SOM represents organic matter content, TN represents total nitrogen content, TP represents total phosphorus content, TK represents total potassium content, AP represents available phosphorus content, AK represents available potassium content, and MC represents soil moisture content. AH stands for alfalfa plant height, AB stands for aboveground biomass, UB stands for underground biomass. *Indicates P ≤ 0.05, ** indicates P ≤ 0.01.

The phylum level analysis revealed a significant positive correlation between TK and *Actinobacteriota*. Additionally, there was a notable positive correlation between MC and *Acidobacteriota*, *unclassified_k__norank_d__Bacteria*, and *Methylomirabilota*. Furthermore, the abundance of *Acidobacteriota* exhibited a significant positive correlation with the growth indicators AH, chlorophyll, AB, and UB in alfalfa. The increment in the abundance of *Acidobacteriota* promoted the growth of alfalfa (Fig. 9a). At the genus level, all species can be divided into two categories: those below *Nocardioides* that have no significant correlation with the indicators were classified into one category, and those above *Nocardioides* that have a significant positive correlation with the indicators were classified into one category (Fig. 9b). The presence of *norank_f__norank_o__Vicinamibacterales* was found to have a significant positive correlation with MC and AH. Similarly, *norank_f__Vicinamibacteraceae* exhibited a significant positive correlation with MC, AH, chlorophyll, AB, and UB. On the other hand, *norank_f__norank_o__norank_c__KD4-96* showed a significant positive correlation with AH, chlorophyll, and UB. Additionally, *norank_f__norank_o__norank_c__MB-A2-108* was positively correlated with MC, AH, and chlorophyll. The presence of *norank_f__Gemmatimonadaceae* was significantly positively correlated with MC and SOM. Lastly, the *Gaiella* bacteria, belonging to the phylum *Actinobacteriota*, was significantly positively correlated with chlorophyll, TK, TN, AP, and AK.

## Discussion

Under sandy soil conditions, the results showed a significant increment in SOM, TN, TP, TK and AP levels when γ-polyglutamic acid was applied. This may be attributed to the effective enhancement of SOM and nutrient levels by γ-polyglutamic acid^23^. As an organic compound, γ-polyglutamic acid exhibits high biodegradability and can be swiftly decomposed into organic matter. Additionally, essential nutrients like nitrogen, phosphorus, and potassium are released during the degradation process, thereby supplying plants with the necessary nutrients^24^. Compared with CK, the sandy soil supplemented with γ-polyglutamic acid exhibited improved water retention, creating more favorable conditions for crop growth. Additionally, γ-polyglutamic acid helped regulate soil pH, enhancing its suitability for plant growth. Previous research, both domestic and international, has shown that the addition of γ-polyglutamic acid can enhance SOM and soil structure, leading to improved soil fertility and water retention^25–27^. This aligns with our study, which observed increased SOM, TP, and TK contents. The decrease in pH value may be due to the acidic byproducts produced during the breakdown of γ-polyglutamic acid, consistent with findings in existing literature^28^.

Alfalfa is an important forage crop that plays a key role in soil improvement and conservation of soil and water resources. Research has shown that the application of γ-polyglutamic acid can significantly boost crop growth, resulting in higher plant biomass and enhanced root structure^29^. These findings align with the observations of alfalfa growth under the G2 treatment in our study. Furthermore, existing literature also suggests that the application of γ-polyglutamic acid can enhance crop yield and optimize nutrient utilization^6^. The enhancement of soil structure through soil improvement may contribute to improved water and nutrient retention, creating a more conducive environment for alfalfa growth^15^. The amino acids in γ-polyglutamic acid exhibit strong ion exchange capabilities, facilitating the release of nutrients adsorbed on soil particles by interacting with surface ions^30^. This ion exchange process also helps to increase the amount of exchangeable nutrients in the soil, making it easier for plants to absorb.

The bacterial community plays a vital role in the microecology of the rhizosphere, contributing greatly to soil nutrient activation and disease control. Several studies have investigated ways to manipulate the structure and diversity of the bacterial community, such as through direct inoculation of growth-promoting bacteria or the use of organic fertilizer^31,32^. Several studies have focused on the effects of γ-polyglutamic acid on soil microbial communities, suggesting its ability to improve the quantity, variety, and evenness of microorganisms^28,33^. These results were consistent with the alterations in bacterial community similarity and diversity index noted in our own research. Our study found that the presence of γ-polyglutamic acid notably influenced the makeup of soil microorganisms. Specifically, there were notable changes in the relative abundance of *Actinobacteriota*, *Proteobacteria*, *Chloroflexi*, *Acidobacteriota*, and *Gemmatimonadota*. Previous research has shown that *Actinobacteriota* was involved in the production of antibiotics and the conversion of challenging substances like cellulose and chitin into organic matter^34^. The results of this study indicate that the relative abundance of *Actinobacteriota* increased with G1 and G3 treatments, while remaining stable with G2 treatments. These findings suggest that the influence of γ-polyglutamic acid on soil organic matter may be concentration-dependent. This study examined the diversity indices of bacterial communities across various treatments and observed variations. These diversity indices have the potential to reflect the functional diversity of bacterial communities under different conditions. Particularly, the G2 treatment demonstrated an enhancement in bacterial diversity and evenness, possibly due to its beneficial impact on soil. This treatment has the capacity to increase the abundance and equilibrium of microbial communities, thus aiding in the rehabilitation of soil ecosystems.

The relationship between soil properties and microbial communities is a topic that requires further investigation in this study. Previous research has shown that the organic matter content significantly affects the diversity of soil flora^35–37^. This study revealed significant correlations between soil properties (SOM, TN, TK, MC, pH) and the community composition of *Proteobacteria*, *Actinobacteriota*, and *Chloroflexi*. These findings suggest that changes in soil properties may be the primary driver of adjustments in microbial community structure. Furthermore, at the genus level, the relative abundance of key bacterial genera, such as *Arthrobacter*, *Nocardioides*, and *norank_f__JG30-KF-CM45*, exhibited significant changes under different treatments. These genera are likely to have crucial functional roles in soil ecosystems, including organic decomposition and nitrogen cycling. Moreover, their relative abundance appears to be closely linked to soil properties, indicating that their influence on soil function may be tied to changes in soil indicators. Moving forward, it is essential to delve deeper into the functional characteristics and metabolic activities of bacterial communities.

## Conclusions

The addition of γ-polyglutamic acid notably enhanced the physical and chemical properties of aeolian sandy soil, increasing the levels of soil organic matter, nitrogen, phosphorus, potassium, and other nutrients. This resulted in improved water retention capacity of the soil. Furthermore, the presence of γ-polyglutamic acid significantly influenced the growth of alfalfa, with the G2 treatment showing a substantial increase in plant height, chlorophyll levels, above-ground and underground biomass of alfalfa, yielding superior results. The use of γ-polyglutamic acid had a significant impact on the soil bacterial community, leading to changes in relative abundance at the phylum level, notably increasing *Actinobacteriota*. Bacterial diversity and uniformity were enhanced with G2 treatment, possibly due to higher levels of soil organic matter, soil water content, and soil total potassium content.

## Data Availability

The datasets generated and/or analysed during the current study are available in the [INSDC] repository, and the sequencing data is available at NCBI (SRA): PRJNA1059744.
